# Detection of Influenza A viruses at migratory bird stopover sites in Michigan, USA

**DOI:** 10.1080/20008686.2018.1474709

**Published:** 2018-05-18

**Authors:** Todd M. Lickfett, Erica Clark, Thomas M. Gehring, Elizabeth W. Alm

**Affiliations:** a Department of Biology and Institute for Great Lakes Research, Central Michigan University, Mount Pleasant, MI, USA; b Region 6 Ecological Services, U.S. Fish and Wildlife Service, Lakewood, CO, USA; c Silver Spring, MD, USA

**Keywords:** Environmental monitoring, Influenza virus, migratory bird, migratory stopover, surveillance

## Abstract

**Introduction:** Influenza A viruses have the potential to cause devastating illness in humans and domestic poultry. Wild birds are the natural reservoirs of Influenza A viruses and migratory birds are implicated in their global dissemination. High concentrations of this virus are excreted in the faeces of infected birds and faecal contamination of shared aquatic habitats can lead to indirect transmission among birds via the faecal-oral route. The role of migratory birds in the spread of avian influenza has led to large-scale surveillance efforts of circulating avian influenza viruses through direct sampling of live and dead wild birds. Environmental monitoring of bird habitats using molecular detection methods may provide additional information on the persistence of influenza virus at migratory stopover sites distributed across large spatial scales.

**Materials and methods:** In the current study, faecal and water samples were collected at migratory stopover sites and evaluated for Influenza A by real-time quantitative reverse transcriptase PCR.

**Results and Discussion:** This study found that Influenza A was detected at 53% of the evaluated stopover sites, and 7% and 4.8% of the faecal and water samples, respectively, tested positive for Influenza A virus.

**Conclusion:** Environmental monitoring detected Influenza A at stopover sites used by migratory birds.

## Introduction

Avian influenza viruses (AIV) are globally distributed in both domestic and wild birds. Influenza A type viruses with all subtypes of the 16 hemagglutinin (H) and nine neuraminidase (N), in most combinations, have been isolated from birds [1,2]. Based on their ability to cause disease in chickens, the AIV are further subdivided into low pathogenicity (LPAI) or high pathogenicity (HPAI) subtypes. The LPAI, which generally produce asymptomatic infections in wild birds, are far more numerous. These LPAI infect more than 105 species of birds from 26 families, but the Anseriformes (ducks, geese, swans) and Charadriiformes (shorebirds) are the primary biotic reservoirs [3–5]. Within some hemagglutinin subtypes, such as H5 and H7, mutation can increase the pathogenic potential [6,7] resulting in disease outbreaks. The potential for HPAI to develop from LPAI poses serious risks to both the commercial poultry industry and to human health. Thus, the ecology of LPAI and their persistence in natural host systems is of interest [2,8].

Avian influenza viruses replicate in the trachea and in the intestines of infected birds [9] and the oropharynx and cloacae may contain high concentrations of virus [9,10]. Feces shed from infected birds also harbor virus that can maintain infectivity in the environment for long periods [9,11,12]. The virus can be transmitted directly between birds or can be acquired by ingestion of contaminated water [10,13,14].

The outbreak of H5N1 in wild birds at Lake Qinghai, China in 2005 highlighted the role of wild migratory birds in global dissemination of HPAIV [15–18]. This recognition spurred investigation of AIV ecology and epidemiology at breeding and wintering sites and along the migratory flyways. Studies revealed that AIV persist in the environment of wild birds. Environmental persistence may enable short- and long-term maintenance of the virus by providing mechanisms for transmission between spatially or temporally separated bird populations [11,19–21], and environmental transmission is crucial for maintaining infection [21–24].

Active AIV surveillance programs have predominantly used live-captured or hunter-killed birds for AIV testing [25], however this is costly and time-consuming [26]. Use of live-captured or hunter-killed birds may also restrict AIV surveillance to specific times of the year (*e.g*., during wing molt). Passive surveillance of the environment used by birds (water and/or deposited feces) would allow monitoring across larger spatial scales and throughout the year. Environmental sites that test positive for AIV could then be targeted for more intensive surveillance under the guidance of a One Health perspective, which might include the evaluation of captured and/or hunter-killed birds and monitoring human and domestic animal health sectors [27,28].

We monitored multiple, migratory stopover sites across the Lower Peninsula of Michigan, USA for Influenza A viruses, by screening fecal deposits and environmental water via real-time quantitative reverse transcriptase polymerase chain reaction (qRT-PCR). This study found that influenza viruses were widely distributed in water and/or feces, with samples from more than 50% of migratory stopover sites testing positive for Influenza A.

## Materials and methods

### Sample collection sites

**Figure 1.**
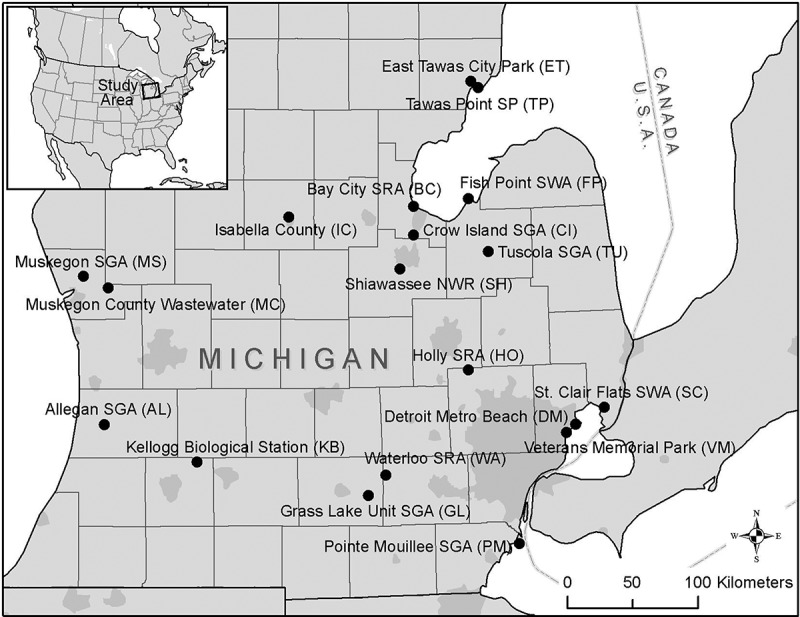
Environmental sampling sites. Locations of sampling sites in the lower-peninsula of Michigan, USA.

Samples of water, ice, and feces were collected from 19 study sites distributed across the southern Lower Peninsula of Michigan, USA (Figure 1, S1 Table), where the Atlantic and Mississippi Flyways intersect [29]. The study sites included natural and constructed lakes, wetlands, and other surface water features used by resident and migrating aquatic birds, and public recreation sites. When possible, site managers were consulted to help identify areas suitable for sample collection at each site. Locations frequently used by large numbers of aquatic birds were considered most suitable, since it was expected that Influenza A virus would be most abundant in such sites. Sites that could be accessed by motor vehicle or walking were preferred due to ease of access for collection. Observations of waterfowl use at the time of sample collection guided specific sampling locations during each sampling trip.

### Sample collection

Water, ice, and fecal samples were collected throughout eight, 3-week sampling periods between February 2007 and April 2008. These sampling periods corresponded to seasonal waterfowl migration events. Samples were collected during the following periods: two periods during spring migration 2007 (Feb/Mar and Apr/May), five periods fall 2007 (Aug/Sep, Sep/Oct, Oct/Nov, Nov, Dec/Jan), and one period spring 2008 (Mar/Apr). Every site was sampled during at least five of the eight 3-week sampling periods. Exceptions for this sampling regime include Detroit Metro Beach Metropark (sampled during four periods), and Grass Lake Unit State Game Area, Tuscola State Game Area, and Veterans Memorial Park (sampled during one period only).

Water samples (300–400 mL or 2 L, depending on protocol) were collected approximately 1 meter from the shoreline of each study location using sterile Whirl-Pak® bags (Nasco International, Fort Atkinson, Wisconsin) and a sample grabber. To help evaluate the effectiveness of the cation-coated filter method (described below), a 40 mL portion of each water sample was aliquoted for comparison of Influenza A detection in un-filtered versus filtered samples. When ice formation prevented the collection of water, ice chunks were obtained using sterile instruments, and placed in Whirl-Pak bags. The ice thawed in the lab, and the melted ice water was then processed as for other water samples. During each site visit, surface water temperature was measured using a red-alcohol thermometer.

Fecal samples were collected at study sites whenever fresh (still moist) bird droppings were encountered during water sample collection. The determination of the likely origin of bird fecal samples was based on physical observations and known migratory bird species in the region, including Canada goose, gull, and duck (multiple species). Each fecal sample was categorized based on the morphology (size, color, composition) of the feces and the context in which the feces were found relative to birds present in the collection area. Fresh fecal material was collected on sterile Dacron swabs (Fitzco Inc., Spring Park, Minnesota), which were immediately placed into tubes containing 1 mL of Viral Transport Media (VTM – 1:1 PBS (phosphate buffered saline) -glycerol supplemented with penicillin G (2 × 10^6^ U/L), streptomycin (200 mg/L), polymyxin B (2 × 10^6^ U/L), gentamicin (250 mg/L), nystatin (0.5 × 10^6^ U/L), ofloxacin HCl (60 mg/L), and sulfamethoxazole (0.2 g/L) and 1% (w/v) final concentration BSA) [30]. Environmental samples were stored on ice during transport to the laboratory. All samples were processed within 48 hours of collection, with the exception of water samples collected in spring of 2007, which were stored for 3–6 months at −20°C prior to processing.

#### Filter-concentration of water and melted ice water samples

Water and melted ice water samples were filtered through cation charged filters (CCF). Two sample volume ranges were used in this study: 1) small volumes of 300–400 mL and 2) large volumes of 2 L. Negatively-charged 0.45 μm pore size HA filters (Millipore Corporation, Billerica, Massachusetts) of either 47 mm diameter (for 300–400 mL samples) or 90 mm diameter (for 2 L samples) were placed onto a vacuum filter apparatus, and 5 mL of 250 mM AlCl_3_ were passed through the membrane to form Al_3_^+^ charged filters [31,32]. Water samples were then vacuum filtered at a maximum filtration rate of 50 mL/min. After the water samples were filtered, a 0.5 mM H_2_SO_4_, pH 3.0 (100 mL or 200 mL, respective to initial sample volume) acid rinse was performed to remove the aluminum ions from the membrane and promote viral recovery. Viruses bound to the membrane were eluted from the filter using either 5 mL or 10 mL of 1.0 mM NaOH (pH 10.8). Elute from the CCF was recovered and neutralized in a sterile 1.7 mL tube containing either 25 μL or 50 µL of 100 mM H_2_SO_4_ (pH 1.0) and 50 μL or 100 µL of 100× Tris-EDTA buffer (1.0 M Tris-HCl [pH 8.0] and 0.1 M EDTA). The concentrated sample resulting from filtration of an initial 2 L sample was placed immediately into an Amicon® Ultra-15 Centrifugal Filter Unit (Millipore Corporation, Billerica, Massachusetts) for ultrafiltration. Each Centrifugal Filter Unit (CFU) was spun at 3,000 rpm (1,300 × *g*) in a Sorvall RT6000 centrifuge until the retentate volume was less than 0.5 mL. Virus-rich solutions were then either processed immediately (as described below) or stored at −20°C until RNA extraction.

#### Faecal samples

Prior to RNA extraction, fecal samples were centrifuged to reduce possible agents inhibitory to subsequent qRT-PCR reactions [33]. Thawed fecal samples were thoroughly vortexed for 5 min, then centrifuged at 4,000 × *g* for 20 min. The clarified supernatant was then transferred to a new sterile 1.7 mL tube and stored at −20°C until RNA extraction.

### Real time quantitative reverse transcriptase PCR

#### RNA extraction

Total RNA was extracted from all sample types (water, ice water, and faeces) using QIAamp® Viral RNA Mini kits (Qiagen, Germantown, Maryland), according to the manufacturer’s instructions. Briefly, 140 µL of sample was mixed with the provided lysis buffer and incubated for 10 min at room temperature. After adding 560 µL of 200 proof molecular grade ethanol (Sigma-Aldrich Corporation, St. Louis, Missouri), the sample was loaded onto the QIAamp spin column membrane and washed using two different wash buffers. Finally, viral RNA was eluted in 60 µL of the provided RNAase-free buffer, and was stored at −20°C until use in RT-qRT-PCR.

#### Detection of influenza a matrix gene

Detection of influenza RNA was accomplished using a fluorogenic probe and primers targeting a highly-conserved region of the Influenza type A matrix gene [34,35] (Table 1). The fluorogenic probe was labeled with 6-carboxyfluorescein (FAM) on the 5ʹ end and a non-fluorescent quencher and minor groove binder (MGB) on the 3ʹ end. Total length of the amplified fragment was 195 base pairs.

**Table 1.** Nucleotide sequences and gene target nucleotide position of the primers and probe used for Real-Time qRT-PCR amplification of the Influenza A matrix gene.

Primer	Nucleotide Sequence (5ʹ® 3ʹ)	Gene target nucleotide position
Forward	TAACCGAGGTCGAAACGTA	36–54
Reverse	GCACGGTGAGCGTGAA	215–230
Probe	TCAGGCCCCCTCAAAGC	74–90

The fluorogenic probe was labeled with 6-carboxyfluorescein (FAM) on the 5ʹ end and a non-fluorescent quencher and minor groove binder (MGB) on the 3ʹ end. Total length of the amplified fragment was 195 base pairs.

Real-Time qRT-PCR reactions were performed on an ABI 7500 Real-Time PCR System (Applied Biosystems, Inc., Foster City, California) using RNA UltraSense™ One-Step Quantitative RT-PCR kits (Invitrogen Corporation, Carlsbad, California). Each 25-µL reaction volume contained 2.5 µL of extracted RNA, 1X RNA UltraSense Reaction Mix, 1.25 µL UltraSense Enzyme Mix, 0.4 µM of each primer, and 0.24 µM of the fluorogenic probe. Thermal cycling conditions were as follows: a single cycle of reverse transcription for 30 min at 45°C, 2 min at 95°C for reverse transcriptase inactivation, denaturing of the RNA/cDNA hybrid, and *Taq* DNA polymerase activation, followed by 50 amplification cycles of 95°C for 5 sec (denaturation) and 60°C for 30 sec (extension). Positive controls for calculation of standard curves were prepared from ten-fold serial dilutions of 1027-bp matrix gene cRNA control templates supplied by Longhorn Vaccines & Diagnostics (San Antonio, Texas). Two or more no template (negative) control reactions were also included in each experiment.

LinRegPCR 12.2 software (Heart Failure Research Center, Amsterdam, Netherlands) was used to establish baseline fluorescence for raw data imported from the 7000 System Sequence Detection Software 1.3 (Applied Biosystems, Inc., Foster City, California). LinRegPCR determines per-sample baseline fluorescence by reconstructing the log-linear phase of PCR reactions, which provides a more robust estimate of baseline fluorescence than algorithms that derive a linear trend from early amplification cycles [36]. Raw sample data that exhibited positive amplification (defined as 7-fold increase in fluorescence) were manually baseline corrected. At least two replicate reactions were run for each sample, and samples showing positive amplification in two or more individual reactions were considered positive for the Influenza A matrix gene.

#### Quantification of influenza a matrix gene

Influenza RNA in environmental samples was quantified using a modified standard curve-based method, the *Cy*_0_ method [37]. Unlike the cycle-threshold (*Ct*) method, the *Cy*_0_ method does not require the assumption of uniform reaction efficiency between standards and unknowns, making it ideal for the quantification of environmental samples that are likely to contain PCR-inhibiting compounds [37]. The *Cy*_0_ value is the intersection point between the abscissa axis and tangent of the inflection point of the Richards curve obtained by the non-linear regression of raw data [37]. As with *Ct*, a smaller *Cy*_0_ indicates a higher number of genome copies, or target virus, in the sample.

To determine the *Cy*_0_ for each positive reaction, STATISTICA 9.1 software (StatSoft, Inc., Tulsa, Oklahoma) was first used to fit the 5-parameter Richards function to baseline corrected fluorescence data using the Levenberg-Marquardt method of unweighted least squares estimation [38–42].

A standard curve was prepared for each experiment from fluorescence data collected for dilutions of the matrix gene cRNA control template. Duplicate reactions from a minimum of three dilutions over a four-log10 range were used in constructing each standard curve. The *Cy*_0_ values from control reactions were plotted as a function of the log10 concentration of control template, and least squares linear regression was used to fit the resulting trend line. The regression models were then used to estimate starting concentrations of each influenza positive environmental sample.

## Results

### Influenza a was detectable in water collected at stopover sites

A total of 210 water and ice samples collected during multiple visits per site were tested, of which 10 were positive for the Influenza A matrix gene (4.8%). The positive water samples were collected at six sample sites widely distributed across Michigan’s Lower Peninsula. Sites included: Kellogg Biological Station (KB), Muskegon County Wastewater (MC), Shiawassee National Wildlife Refuge (SH), St. Clair Flats State Wildlife Area (SC), Tawas Point State Park (TP), and Waterloo State Recreation Area (WA) (Table 2).

**Table 2.** Distribution of water samples testing positive for the Influenza A matrix gene across 19 locations in the Lower Peninsula of Michigan, USA.

Site Code	*n*	Positive (no.)	Positive (%)
AL	18	0	0
BC	6	0	0
CI	14	0	0
DM	2	0	0
ET	3	0	0
FP	14	0	0
GL	4	0	0
HO	13	0	0
IC	11	0	0
KB	14	1	7
MC	13	2	15
MS	20	0	0
PM	22	0	0
SH	17	2	12
SC	17	1	6
TP	5	3	60
TU	1	0	0
VM	2	0	0
WA	14	1	7
Total	210	10	5

Influenza A positive water samples were identified in four of the three-week sampling periods, with the majority of positive samples detected during fall migration. One positive water sample was collected at Waterloo State Recreation Area during spring migration 2007 (n = 52, 1.9%), nine during fall migration (n = 131, 6.9%), and no positives were identified during spring migration 2008 (n = 27). Of the nine positive water samples collected during fall migration, three came from the same shallow, temporary pool behind the beach berm crest at Tawas Point State Park, collected on three separate dates spanning one month (8/23/07, 9/4/07, and 9/23/07). The other six positives were collected from late October to early January, two from Muskegon County Wastewater (10/29/07 and 11/25/07), two from Shiawassee NWR (11/7/07 and 11/30/07), and one each from St. Clair Flats SWA (11/19/07) and Kellogg Biological Station (1/2/08). Mean water temperature recorded at sampling sites varied from a high of 24.9°C in Aug/Sep 2007, to a low of 0.5°C in Dec/Jan 2007–2008, and pH across all sites ranged from 6.0 to 7.0.

The CCF method for concentrating water samples was used for 194 of the 210 water samples. Of these, unfiltered aliquots were directly compared to CCF-concentrated aliquots of the same water for 114 samples, which included six of the 10 positive samples (Table 3). Two water samples tested positive for the Influenza A matrix gene in both neat water samples and CCF concentrated samples, however, three samples tested positive only following CCF concentration. One sample was positive in the unfiltered aliquot but was not positive following CCF concentration. The reason for this is unknown, but target degradation during CCF processing may have occurred. One additional sample, WA-02, was positive using the CCF method, but an unfiltered aliquot of the sample was not available for comparison.

**Table 3.** Quantity of Influenza A matrix gene molecules present in replicate reactions of select water samples analyzed using the *Cy*_0_ method. Samples were unfiltered or were concentrated using the cation-coated filter (CCF) method.

				Molecules/reaction	
Filtration method	Collection date	Sample	Positive reactions (no.)	*x*	SD
Unfiltered (n = 114)	11/19/07	SC-13	2	3.7	1.5
	11/25/07	MC-21	2	0.1	0.0
	11/30/07	SH-11	1	0.2	
CCF (n = 194)	4/7/07	WA-02	2	169.8	211.4
	10/29/07	MC-19	2	3.8	1.7
	11/7/07	SH-08	2	6.9	1.3
	11/25/07	MC-21	2	290.7	31.5
	11/30/07	SH-11	1	5.7	
	1/2/08	KB-11	3	18.1	6.2

Concentrated water samples from Muskegon County Wastewater (MC-21) and Waterloo SRA (WA-02) contained the most Influenza A RNA with 290.7 (SD = 31.5) and 169.8 (SD = 211.4) molecules per reaction, respectively (Table 3). Sample SC-13 from the St. Clair Flats SWA had the highest average molecules per reaction of the water samples that were unfiltered (*x* = 3.7, SD = 1.5). Samples SH-11 from Shiawassee NWR and MC-21 tested positive for Influenza A both before and after use of the CCF method, but estimates of molecules per reaction were higher for both samples after use of the CCF. Overall, the mean and median number of molecules detected per reaction were greater in samples concentrated with the CCF, though statistical significance could not be established due to small sample size. The mean *Cy*_0_ was 38.9 cycles (SD = 2.9) for all concentrated samples and 45.1 cycles (SD = 2.4) for unfiltered water samples.

### Influenza a was detectable in fecal samples collected at stopover sites

Two hundred sixty-four fecal samples collected at sites across the study area were tested, of which 19 were positive for the Influenza A matrix gene (7.2%). Positive fecal samples were collected at the following six sampling sites: Bay City State Recreation Area (BC), Detroit Metro Beach Metropark (DM), East Tawas City Park (ET), Shiawassee National Wildlife Refuge (SH), Tawas Point State Park (TP), and Veterans Memorial Park (VM) (Table 4). No positive fecal samples were recovered during spring migration 2007 (n = 4) and only one positive fecal sample was detected during spring migration 2008 (n = 91, 1.1%). Eighteen positive fecal samples were collected during the fall migration (n = 169, 10.7%); 15 in Aug/Sep 2007 and three in Oct/Nov 2007. Of fecal samples tested, SH-37 collected from Shiawassee in April 2008 had the highest estimated mean molecules per reaction (*x* = 30,302, SD = 386; Table 5). The mean *Cy*_0_ reported for positive fecal samples was 34.0 cycles (SD = 7.0), and all positive samples examined had a *Cy*_0_ less than 40.

**Table 4.** Distribution of faecal samples testing positive for the Influenza A matrix gene across 9 locations in the Lower Peninsula of Michigan, USA.

Site Code	*n*	Positive (no.)	Positive (%)
BC	2	2	100
DM	21	3	14
ET	19	4	21
KB	78	0	0
MC	40	0	0
MS	8	0	0
SH	69	3	4
TP	17	6	35
VM	4	1	25
Total	264	19	7

**Table 5.** Quantity of Influenza A matrix gene molecules present in replicate reactions of select fecal samples analyzed using the *Cy*_0_ method.

Collection date	Sample	Positive reactions (no.)	Molecules/reaction	
			*x*	SD
11/7/07	SH-07	2	32	6
11/7/07	SH-08	2	51	7
4/3/08	SH-37	2	30,302	386

### Analysis of both water and faecal samples may be necessary to identify sites impacted by AIV

Over the course of this study, fecal and water samples were collected from the same site on the same date 16 times. On two collections, both fecal and water samples tested positive (Tawas Point 9/4/07 and Shiawassee 11/7/07). On three sample dates positive fecal samples were recovered while water samples tested negative (Bay City 9/4/07, East Tawas 9/4/07, Shiawassee 4/3/08). On two dates water samples tested positive but the fecal samples collected at the site were negative (Tawas Point 9/23/07 and Kellogg Biological 1/2/08). Nine of these paired collections did not return any samples positive for Influenza A.

## Discussion

Evidence has been accumulating over recent years to suggest that the persistence of Influenza A viruses in the environment may facilitate indirect transmission, which is critical to the epidemiology of influenza infection in wild water birds [22,23]. The presented study demonstrated the use of environmental sampling as an effective method for rapid detection of influenza viruses at migration stopover sites located in Michigan’s Lower Peninsula. Influenza A virus was detected in fecal (7%) and water (5%) samples collected at 10 of the 19 (53%) sites surveyed.

Hinshaw and co-workers [43,44] were the first to report successful isolation of Influenza A viruses from water samples collected from lakes in Alberta, Canada. Since then, other researchers have attempted to isolate or detect avian influenza viruses in environmental samples for the purposes of surveillance. In North America, Influenza A has been detected in water samples collected in Minnesota, Alaska, Oklahoma, and California [20,45–48]. Only 5% of the 210 water samples tested in this study were positive by RT-qRT-PCR for the Influenza A matrix gene. Similar studies have documented higher detection rates in water samples, though reported prevalence varies widely by study, and in some cases with higher detection rates the water samples were collected near trapped sentinel birds or domestic ducks [43–45,49]. The prevalence of influenza positive fecal samples in this study is similar to other studies of environmentally collected fecal samples in North America [20,48,50]. In the present study, a greater percentage of fecal samples returned positive results for Influenza A as compared to water samples. This is consistent with a higher viral load in fecal samples and less dilution effect in fecal material. Since many important waterfowl species (particularly *Anas* spp.) spend most of their time in water, feces are not often deposited on land where they can be sampled. Therefore, water analysis could be a critical component to comprehensive environmental monitoring.

Numerous factors including species, age, sex, and densities of birds at the site affect detection rates of influenza in water and feces [2,5,51,52]. The sites with the greatest number of positive samples were the Muskegon Wastewater Management System, the Shiawassee National Wildlife Refuge, and Tawas Point State Park. These sites are all recognized Important Bird Areas where 250–300 different bird species can be found [53]. These sites are also important stopover sites utilized yearly by tens of thousands of migratory waterfowl [54]. The official fall migration runs from late September to early February, and peak waterfowl numbers occur from the middle of October through the middle of December [54]. In this study, the proportion of water and fecal samples testing positive was higher in those samples recovered during the fall, as opposed to the spring, migration. It is possible that the introduction of a freeze-thaw cycle and extended storage of water samples collected in the spring of 2007 may have caused viral degradation, contributing to the lower detection rate observed in the spring. However, experimental results have demonstrated that detectability of influenza RNA is not substantially altered by limited freeze-thaw cycles or storage within the range of conditions used in this study [55]. Additionally, our findings are consistent with observations that the collection sites visited hosted more birds during fall migration. For example, ~48,000 waterfowl stop at the Shiawassee National Wildlife Refuge during the fall migration but only ~19,000 stop during spring migration [53,54]. Not only are bird densities greater during fall migration, but birds spend more time at stopover sites during fall migration [56]. Stops during fall migration are longer as migratory birds require fuel for subsequent legs of the migration. In addition, juvenile birds participating in their first migration in the fall are not as efficient foragers as adults are, and spend increased time at fall migration stops [56]. AIV is also more prevalent in immunologically-naïve juveniles compared to older birds, which may gain some immunity to AIV through repeated exposure over time [57]. In comparison, the spring migration is more rapid and of shorter duration as mature birds are in competition to reach breeding grounds. Influenza infection rates are also reported to be greater during fall migration and infected birds may remain longer at stopover sites [58]. A 26-year survey of waterfowl in North America showed a 22.2% infection rate during fall migration, but only a 0.3% infection rate in ducks during their return flight northward in the spring [59].

The persistence of virus in feces or water, which is related to temperature, pH, and salinity, also affects detection of influenza in environmental samples [11,12,60]. With the exception of the positive water samples from Tawas Point State Park, water temperatures recorded at sites when positive samples were recovered during the fall of 2007 were moderate and did not exceed 11°C and the average water temperature at all sites dropped to 2.6°C by November. At these temperatures, intact influenza virus could persist, enabling detection of virus particles deposited in the environment by transient waterfowl. Other studies have found increased detection rates in environmental water and fecal samples during fall months [21,45,61].

Not all positive samples were associated with colder water temperatures. The three positive water samples collected at Tawas Point State Park (Aug-Sep) were all collected from a shallow, temporary pool that formed on the beach behind the berm crest. Water temperatures in this pool at the time of collections ranged between 17°C and 19°C. Large numbers of Ring-Billed gulls (*Larus delawarensis*) were present on all three collection dates. The highest prevalence of influenza in gulls has been reported to be late summer and early fall [2]. This corresponds to the samples collected at Tawas Point during this study, which supports the increased attention gulls have received in recent years for a role in influenza epidemiology [62].

Methods used for virus detection also affect determination of influenza prevalence. Detection methods fall in to two broad categories; isolation of virus in embryonated chicken eggs or molecular detection of virus RNA by RT-PCR. Egg cultivation of environmental water was first reported by Hinshaw and co-workers to detect influenza in lakes used by waterfowl in Alberta, Canada [44]. RT-PCR was first used on environmental water samples collected at lakes used by waterfowl in Siberia, Russia [21]. Both direct and concentrated samples have been used with either isolation or molecular detection. It is more likely that influenza virus will be detected when environmental water samples are concentrated [63,64]. Feces from infected birds contain high levels of virus [9] and do not require concentration. In addition to detection, egg isolation may provide information on infectivity. However, egg isolation is slow and expensive, and because cultivation increases the number of infectious virus particles, cultivation may require enhanced biosecurity. Comparatively, RT-PCR is rapid, more sensitive [14], less expensive, and does not increase the amount of virus. However, loss of RNA target or PCR inhibition by organic material in the sample may lead to an under-estimation of virus at the site. In this study, centrifugation was used to clarify fecal samples prior to RT-qRT-PCR, which reduces inhibition [33]. Potential inhibition in both water and fecal samples was also addressed in this study by quantifying using the *Cy*_0_ method [37]. Conversely, RT-PCR may detect fragments from virus particles that have lost infectivity, over-estimating the potential for subsequent transmission of influenza at the site.

The best estimations of the distribution, persistence, and pathogenicity potential of Influenza virus in a particular environment of interest will likely be made using a spatially- and temporally-tiered surveillance approach. Across a larger spatial extent, screening of environmental sites could be made using RT-PCR to screen water and/or feces during fall and spring months, as described in this study. Although individual fecal samples were collected and analyzed in this study, feces could be pooled, which would allow a greater number of fecal samples to be analyzed [65]. If further characterization of influenza positive sites were of interest, the RT-PCR results could be confirmed by virus isolation. Further characterization of subtypes may be warranted, particularly for highly pathogenic subtypes seen in North American migratory birds [5]. If positive samples/subtypes are recovered, more localized and intensive cloacal and/or pharyngeal sampling of hunter-killed or live-captured birds could also be added to provide additional information on infection rates and species involved. To better evaluate the potential for cross-species transfer, environmental monitoring may also be coordinated with other sampling efforts, such as at poultry facilities near migratory bird stopover sites.
