# Supplementary material for: Detection of Influenza A viruses at migratory bird stopover sites in Michigan, USA
Source: Infect Ecol Epidemiol. 2018 May 18;8(1):1474709. doi: 10.1080/20008686.2018.1474709 (PMC5965024; doi:10.1080/20008686.2018.1474709)
Supplement: Supplemental Material [file ZIEE_A_1474709_SM2415.docx]

Supplemental Table 1. Characteristics of Sampling Sites in Michigan

| Site | ID | Location | Administrator |
| --- | --- | --- | --- |
| Allegan  State Game Area | AL | Allegan | Michigan Department of Natural Resources and Environment |
| Bay City  State Recreation Area | BC | Bay City | Michigan Department of Natural Resources and Environment |
| Crow Island  State Game Area | CI | Bay City | Michigan Department of Natural Resources and Environment |
| Detroit Metro Beach Metropark | DM | Harrison Twp | Huron-Clinton Metroparks |
| East Tawas City Park | ET | East Tawas | City of East Tawas |
| Fish Point  State Wildlife Area | FP | Unionville | Michigan Department of Natural Resources and Environment |
| Grass Lake Unit  State Game Area | GL | Grass Lake | Michigan Department of Natural Resources and Environment |
| Holly  State Recreation Area | HO | Holly | Michigan Department of Natural Resources and Environment |
| Isabella County | IC | Mt. Pleasant | Central Motor Sports, Edward Oplinger |
| Kellogg  Biological Station | KB | Hickory Corners | Michigan State University |
| Muskegon County Wastewater | MC | Muskegon | Muskegon County Wastewater Management System |
| Muskegon  State Game Area | MS | Twin Lake | Michigan Department of Natural Resources |
| Pointe Mouillee  State Game Area | PM | Rockwood | Michigan Department of Natural Resources |
| Shiawassee  National Wildlife Refuge | SH | Saginaw | U.S. Fish & Wildlife Service |
| St. Clair Flats  State Wildlife Area | SC | Harsens Island | Michigan Department of Natural Resources |
| Tawas Point  State Park | TP | East Tawas | Michigan Department of Natural Resources |
| Tuscola  State Game Area | TU | Cass City | Michigan Department of Natural Resources |
| Veterans Memorial Park | VM | St. Clair Shores | City of St. Clair Shores |
| Waterloo  State Recreation Area | WA | Grass Lake | Michigan Department of Natural Resources |
